# Mabellini: a genome-wide database for understanding the structural proteome and evaluating prospective antimicrobial targets of the emerging pathogen *Mycobacterium abscessus*

**DOI:** 10.1093/database/baz113

**Published:** 2019-11-13

**Authors:** Marcin J Skwark, Pedro H M Torres, Liviu Copoiu, Bridget Bannerman, R Andres Floto, Tom L Blundell

**Affiliations:** 1 Department of Biochemistry, University of Cambridge, Cambridge CB2 1GA, UK; 2 Molecular Immunity Unit, Department of Medicine University of Cambridge, MRC-Laboratory of Molecular Biology, Cambridge CB2 0QH, UK and; 3 Cambridge Centre for Lung Infection, Royal Papworth Hospital, Cambridge CB23 3RE, UK

## Abstract

*Mycobacterium abscessus,* a rapid growing, multidrug resistant, nontuberculous mycobacteria, can cause a wide range of opportunistic infections, particularly in immunocompromised individuals. *M. abscessus* has emerged as a growing threat to patients with cystic fibrosis, where it causes accelerated inflammatory lung damage, is difficult and sometimes impossible to treat and can prevent safe transplantation. There is therefore an urgent unmet need to develop new therapeutic strategies. The elucidation of the *M. abscessus* genome in 2009 opened a wide range of research possibilities in the field of drug discovery that can be more effectively exploited upon the characterization of the structural proteome. Where there are no experimental structures, we have used the available amino acid sequences to create 3D models of the majority of the remaining proteins that constitute the *M. abscessus* proteome (3394 proteins and over 13 000 models) using a range of up-to-date computational tools, many developed by our own group. The models are freely available for download in an on-line database, together with quality data and functional annotation. Furthermore, we have developed an intuitive and user-friendly web interface (http://www.mabellinidb.science) that enables easy browsing, querying and retrieval of the proteins of interest. We believe that this resource will be of use in evaluating the prospective targets for design of antimicrobial agents and will serve as a cornerstone to support the development of new molecules to treat *M. abscessus* infections.

## Introduction

*Mycobacterium abscessus* is a species of rapid growing mycobacteria (RGM), comprised of three subspecies (1, 2) (*abscessus*, *massiliense* (3) and *boletti* (4)) collectively referred to as *M. abscessus complex* (1).

While most RGM, such as *Mycobacterium smegmatis* and *Mycobacterium marinum,* are saprophytic organisms, either found as free-living organisms or intracellular parasites, mainly infecting amoebae (5), data regarding environmental isolates of *M. abscessus* are scarce (6). In fact, it is one of the few RGM capable of infecting human hosts, causing skin and pulmonary diseases, and is frequently (but not exclusively) isolated from patients with pre-existing underlying diseases, such as cystic fibrosis (CF) (6). For CF patients, *M. abscessus* infections are particularly troublesome, causing an accelerated inflammatory lung damage, evidenced by rapid decline in lung function (7) and frequently preventing safe lung transplantation (8, 9). Unlike other nontuberculous mycobacteria, *M. abscessus* can be transmitted from patient to patient (10), probably through exposure to infectious aerosols and/or fomites (11).

Treating *M. abscessus* infections is extremely challenging due to (i) intrinsic resistance to antibiotics, such as isoniazid, rifampin, pyrazinamide and ethambutol, used to treat *Mycobacterium tuberculosis* (6); (ii) acquired drug resistance, such as resistance to aminoglycosides via mutations in the 16S ribosomal RNA (12, 13); (iii) inducible tolerance, such as in the case of clarithromycin, a macrolide drug, where resistance is likely to occur through the methylation of ribosomal RNA carried out by the erythromycin ribosomal methylase Erm (14, 15, 41); and (iv) survival within antibiotic-inaccessible niches (such as within biofilms) (16). As a consequence, treatment failure is extremely high (60–70% in individuals with CF (17)) with patients experiencing persistent culture positivity, relapse following initial sputum culture conversion (18–20) or early recurrence after the end of treatment (21).

Recently, Luthra et al. (22) have reviewed the genotypic determinants of antibiotic resistance in *M. abscessus* and pinpointed several proteins, which act through distinct mechanisms and are likely responsible for the emergence of resistant phenotypes; we revisit some of this in the results section. Besides antibiotic resistance, other phenotypic aspects related to virulence have been intensively studied in the past decades, but many underlying mechanisms involved in *M. abscessus* pathogenesis are still not completely understood.

*Mycobacterium abscessus* can grow with two distinct colony morphologies, namely smooth (S) and rough (R) phenotypes (23, 24). *Mycobacterium abscessus* isolates with an R morphotype have been associated with increased pathogenicity (25). It was recently shown that the conversion from S to R phenotype can occur during the course of an infection and is caused by loss of glycopeptidolipid (GPL) (24), due to mutations taking place in the GPL locus (26). This is an interesting case in which loss of function leads to selective advantage and, ultimately, to increased pathogenicity.

Another interesting hallmark associated with R isolates (27) is their ability to form cord-like structures, in which the long axes of the bacilli align to the horizontal axis of the cord, a trait shared by several other mycobacterial species (28). It is believed that this kind of organization is directly linked to the pathogenicity, hindering effective phagocytic clearance (29), enhancing the persistence of bacterial cells inside macrophages (28) and causing extensive macrophage damage (30), Llorens-Fons et al. (31) have presented evidence that trehalose polyphleates (TPP) are crucial molecules for the formation of cords and Burbaud et al. (32) have shown that disruption of the TPP synthesis, achieved through the mutation of the polyketide synthase gene (Pks), does not affect GPL production, indicating that cord formation is dependent on several independent processes.

The whole *M. abscessus* genome was released in 2009 (33), allowing for the detailed study of the molecular basis behind these phenotypic traits and virulence factors. Ripoll and colleagues sequenced the complete genome from *M. abscessus* ATCC 19977T strain (S phenotype), using a whole-genome shotgun strategy. Their findings show that its genome comprises a circular chromosome of 5 067 172 base pairs with 4920 predicted coding sequences (EMBL accession number: CU458896) and also a mercury resistance plasmid of 23 kb (EMBL accession number: CU458745), similar to pMM23, found in *M. marinum*.

The authors reported a series of both mycobacterial and non-mycobacterial virulence factors, including the mammalian cell entry operons, phospholipase C and several antibiotic resistance genes. Especially interesting was the extensive amount of horizontally acquired genetic material, exhibiting synteny with a number of different genera, such as *Rhodococcus*, *Pseudomonas* and *Burkholderia* (33). These efforts have been curated in both general databases, like UniProt (34) (Proteome ID: UP000007137), and more specialized ones, like the Mycobrowser (35), which contains annotated genomic data from eight different mycobacterial species, including slow growers like *M. tuberculosis* and *Mycobacterium leprae* and rapid growers, like *M. abscessus* and *M. smegmatis*.

However, structural information regarding *M*. *abscessus* proteins is scarce and, at the time of writing (07/02/2019), only 53 proteins, of which 39 are unique, had their structures determined, corresponding to less than 1% of the reported coding sequences.

Comprehensive understanding of protein function, the effects of mutations and successful structure-guided drug discovery is contingent on availability of accurate molecular models of the target proteins. These may be obtained either through experimental structure determination, or less costly, through computational modelling.

Our group has previously developed a database for the structural proteome of *M. tuberculosis* (36) using a set of in-house tools that rely on well-stablished databases, such as CATH (37) and SCOP (38) to retrieve domain-based information and use it in the modelling of the protein structures.

Besides *M. tuberculosis*, other organisms have had their structural proteomes modelled, many of which are available in the well-known UCSF ModBase (39) but, to our knowledge, such information is not available for *M. abscessus*. Additionally, online databases of protein models suffer from the insufficient computational sampling, since due to a throughput bottleneck, trade-offs are often made in what regards exhaustive structural sampling, an issue we tried to overcome with the approach proposed here.

In this work, we report the development of *Mabellini*, a novel, extensively annotated structural database, in which we focus on acquiring good-quality models for the *M. abscessus* proteome, providing an intuitive web interface, easily accessible to the potential end users, who will be able to navigate through the proteomic data, and inspect and/or download the generated models. Knowledge concerning the protein structures is invaluable for understanding protein function and the effects caused by mutations. We also showcase examples of modelled proteins that are known to be implicated in some of the phenotypic features discussed above.

## Methods

### Protein structural profiles database

The modelling approach used in this work relies on a structural profile database (TOCCATA), which combines the domain-based information present in CATH (37) and SCOP (38) databases with information from primary data sources: the Protein Data Bank (PDB) (40) and UniProt (34). Each profile is annotated with functional information (derived from UniProt) and small-molecule-biding data (derived from PDB) and contains sets of pre-aligned representative structures, clustered by functional state. Each of the structures is annotated with the structural quality metrics, both directly derived from PDB (e.g. R-free, resolution) and those computed, e.g. AEROSPACI (41, 42) score. Due to incomplete annotation of the PDB in the AEROSPACI database, we fill in the missing values using a simple linear model, see Supplementary Material.

By identifying one profile from TOCCATA, our model-building pipeline (hereafter referred to as ‘Vivace’) accesses a large set of well-curated and annotated structures, permitting the identification of often non-obvious mappings between target sequences and template. TOCCATA contains three sets of profiles: single domain, multi domain and unclassified. The first two are based on SCOP and CATH annotations, while the third contains all structures that are covered by neither SCOP nor CATH. Notably, sequences contained in each profile are clustered into groups sharing 95% identity (cluster representatives), in order to avoid including the same protein (or its variants) more than once, thus ensuring sufficient diversity of the profile.

A more detailed description of the TOCCATA architecture can be found in (36).

### Proteome modelling pipeline (Vivace)

**Figure 1:**
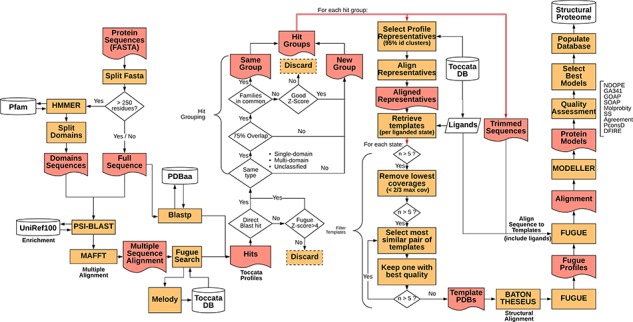
Simplified flowchart of Vivace pipeline execution. Orange boxes represent processes, red nodes represent generated files, white cylinders represent databases and white diamonds represent decision-making nodes. The two red double arrows denote branching steps of the pipeline.

Representative sequences for the proteome of *M. abscessus* retrieved from UniProt (Proteome ID: UP000007137) were organized as individual sequences, each of which formed a starting point for the pipeline. The full procedure for template selection, model generation and scoring are described below and depicted schematically in Figure 1. Thanks to Vivace being built using Ruffus (43), a pipelining solution, each run may progress at its own pace, allowing for optimal use of computational resources. This architecture allows for scaling the method to multiple machines, provided they share a common file system, allowing for use of *ad hoc* cluster solutions.

For each of the identified gene sequences (target sequences) that are longer than 250 residues, we perform domain decomposition, through a HMMER (44) search against PfamA (45) database. As much of TOCCATA is based on single domains, we ensure that we do query each of the putative domains against the profile database. If we were to rely only on the full-length sequence, domains with weaker evolutionary footprint (smaller, less conserved families) could remain undetected.

The full sequence and those of identified domains are subjected to PSI-BLAST searches against the UniRef100 database. The resulting sequences are re-aligned using MAFFT (46) and used for prediction of structural characteristics of the protein (e.g. secondary structure, solvent accessibility, etc.). This information in turn is fed into MELODY (part of the FUGUE (47) suite), a program that produces a compact representation of this information (profile), to be used for querying the TOCCATA database.

Using each of these profiles, Vivace performs FUGUE-based profile–profile search on ‘single-‘ and ‘multi-domain’ subsets of the TOCCATA database. Only when no hit with sufficiently high Z-score (Z-score > = 8) is found, the ‘unclassified’ set is searched. In so doing, we achieve a compromise between search speed and precision, as it can be safely assumed that a significant fraction of proteins in any given proteome comprises well-defined evolutionary domains, described in either SCOP or CATH. It is only when Vivace is unable to identify such domains, that a much more time-consuming search against the set of ‘unclassified’ profiles is launched.

In parallel to the FUGUE search, Vivace conducts a BLAST search against pdbaa (48), a list of the protein sequences in PDB. The resulting hits with sequence identity of at least 70% are located in the TOCCATA database, and each profile that contains this protein or its subset is automatically included in the subsequent processing, even if it has not been detected by FUGUE search. In this way, we ensure that profiles containing very close homologs of the target are always included, even if there are already good hits present, identified from an initial FUGUE search.

Of the identified profiles, the ones with low confidence (FUGUE Z-score below 4.0) are discarded. Profiles retrieved through BLAST searches have no inherent Z-score associated and thus are always retained. All retained profiles undergo a grouping process, in order to reduce redundancy. The profiles are compared and are grouped where they cover an overlapping span of the queried sequences (over 75% overlap) and if they contain at least one SCOP or CATH family in common. Otherwise they form new ‘hit groups’. Each group is then further processed, and the query sequences are trimmed to match the length of the selected profiles, inside which the potential template structures reside. This step results on average in three hit groups per protein with 95% of the protein targets having at most seven hit groups (see Supplementary Figure S1).

In each of the ‘hit groups’, Vivace retains only the profile clusters (cf. TOCCATA section) whose representative sequence presents no less than 20% identity to the query in comparison to the best one. However, if the cluster to be discarded affords a higher coverage of the query sequence and has at least 50% sequence identity, it is retained. This step permits pruning the template structures that may not be appropriate for successful modelling (preventing undue bias), while ensuring the highest possible coverage of the query by the templates.

In each of the selected cluster profiles, the potential templates are classified into functional states, with respect to the bound ligands and cofactors. There is also an ‘apo’ state, denoting templates that are devoid of ligands, as well as any state, which contains all the templates available in the cluster. This step ensures that each of the ‘states’ comprises only structurally compatible, superimposable templates, making them well suited for modelling.

For each of the states, Vivace selects up to five templates for modelling. Initially, while there are still more than five candidates, the ones with low-coverage (less than two-thirds the maximum coverage) are iteratively removed. Then, the algorithm computes an all-against-all sequence identity matrix of all the templates in the state. The template set is then iteratively pruned, in a bottom-up approach, starting with the most mutually similar template pair and rejecting the one that presents the lower crystallographic quality (as measured by AEROSPACI scores present in TOCCATA database). This process is repeated until at most five templates per state remain, thus ensuring maximal diversity of template structures in terms of amino acid sequence, while maximizing their expected quality.

The selected templates in each of the states are re-aligned to the target sequence using BATON (manuscript in preparation), a modified open implementation of the COMPARER method (49) for protein structure alignment beyond rigid body superposition. This step allows slight adjustment to the pre-computed alignment present in TOCCATA, optimizing the fit between proteins involved in modelling, thus prospectively facilitating the subsequent model construction.

Given the BATON alignment of the selected templates in each of the states for each hit group in a protein, Vivace proceeds to the comparative modelling stage, using MODELLER (50)—a macromolecular modelling suite, originally developed within the group. Using the ‘loopmodel’ protocol implemented in MODELLER, we produce three models with two candidate loop realizations each. All the models are subject to the *very_fast* refinement protocol that is available in MODELLER suite. In our experience, increasing computational effort for refinement or producing greater number of models per state do not render substantially more useful models. Models are assessed with DOPE (51) and GA341 (52) potentials.

### Model quality assessment and selection

Modelling protocol renders tens (up to thousands, contingent on the number of available profile hits and states) of models per target, some of which are of substandard quality or very similar to the others. The experience of CHOPIN demonstrates that the end user prefers a limited number of models that help them explain the biological questions, rather than a comprehensive ensemble, which is often perceived as overwhelming. Thus, we have opted for displaying an ensemble of up to five models ranked according to their quality, as described below.

The first post-modelling processing stage consists of three different filtering steps for the removal of models exhibiting (i) extensive main chain clashes, (ii) large poorly resolved loops and (iii) loosely interacting ligands.

Then, to provide a meaningful model ensemble, we devised an algorithm to select models that (i) are in a not yet displayed functional state (liganded, different conformation), (ii) cover a not yet displayed span of the protein or (iii) model a region of the protein with better accuracy than models already displayed.

To do so, we define two auxiliary quality metrics Q1 and Q2. The first metric (Q1) implicitly corresponds to global quality of the protein and is a linear combination of 80% structural consensus between models (computed by PconsD (53), via the comparison of residue–residue distance matrices, thus allowing for conformational flexibility in the comparison) and 20% stereochemical quality (computed by MolProbity, (54) in terms of MolProbity percentage score). The other metric (Q2) is a linear combination of GOAP-AG (55), GOAP-score and SOAP (56) potentials, scaled by reciprocal of the square of number of residues in the model. To avoid selecting incorrectly folded proteins and to discriminate between decoys with similar energy levels, we amend the Q2 metric with PconsD score at the same weight as each of the knowledge-based potentials (Supplementary Figure S2).

Each of the constituent factors is expressed in terms of percentile across all the produced models. Therefore, a model with a Q1 or Q2 scores of 0.7 is explicitly better, in terms of this metric, than 70% of other models evaluated. High Q scores indicate that there is a significant difference in quality between models in the ensemble, and hence the high-scoring models are much more likely to be correct, occupying low energy minimum for a given sequence. On the other hand—low scores do not necessarily indicate poor models, but rather models in an ensemble lacking structural consensus (in the case of Q1) or exhibiting relatively shallow free energy wells.

### Gene Ontology annotation

Research groups are often interested in the biological functions of genes and in specific subsets thereof. Therefore, we constructed a clear, intuitive and interactive navigation tool that enables quick access to subsets of genes. For that, we implemented a ‘sunburst’ representation of the Gene Ontology (GO) terms with which *M. abscessus* proteins have been annotated.

Ontologies are sets of well-defined terms and relationships that represent the knowledge for and within a given domain. As such, the GO classification (57) actually relies on a complex set of relationships between the component terms, which do not form a simple hierarchical tree.

There are six basic relationships between GO terms: (i) *is_a*, (ii) *part_of*, (iii) *has_part*, (iv) *regulates*, (v) *positively_regulates* and (vi) *negatively_regulates*. Even considering solely the *is_a* relationship, the GO is still more complex than a simple tree, in the sense that a child node may have more the one parent node. Therefore, the GO network can be defined as a directed acyclic graph (58), since there are no cycles, i.e. under no circumstance a node (GO term) can be a parent of itself. It is also unbalanced (different branches have variable depths) and ragged (there is actually no clear concept of ‘level’ or ‘hierarchy’). Furthermore, it is worth noting that the GO terms are divided into three independent domains that are ‘not’ connected by any ‘is a’ relationship: (i) molecular function, (ii) biological process and (iii) cellular component (57).

**Figure 2:**
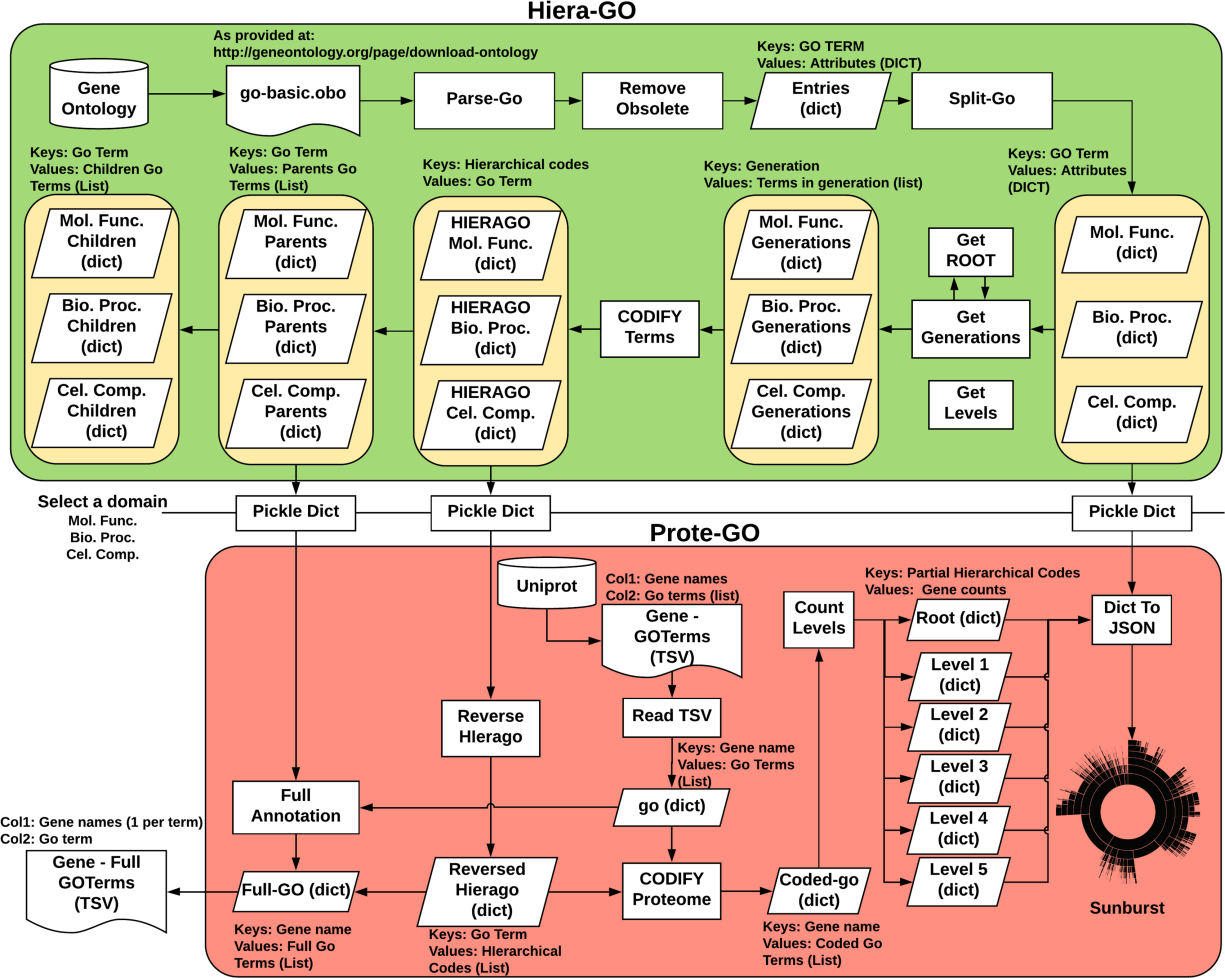
Algorithm used to classify *M. abscessus* Gene Ontology terms as an interactive sunburst graph. The first set of functions was termed Hiera-GO and creates a set of codified GO-term dictionaries, one for each domain. The second set of functions was termed Prote-GO and uses the created dictionaries to produce the sunburst data and the complete GO annotation for each *M. abscessus* gene.

We devised an algorithm (Hiera-GO, Figure 2, top box) that could use the parent–children relationships between the terms to create a simple hierarchical architecture. For this purpose, we used the *go_basic.obo* ontology, available at: http://geneontology.org/page/download-ontology, and parsed it to create a set of dictionaries, one for each domain (i.e. namespace) with the GO term as a key and the term attributes (*id, name, namespace, is_obsolete, is_a*) as an inner dictionary. We then used the *is_a* relationships to deduce the generations and to codify the terms, ultimately assigning a set of codes to each one of the terms, where each assigned code refers to a path through which that term can be accessed from the root node (Supplementary Figure S3).

In a second step (Prote-GO, Figure 2, bottom box), the codified terms were used to construct a hierarchical representation of *M. abscessus* proteome annotation retrieved from Uniprot. Subsequently, this hierarchical representation was written to a JSON file, which was adopted in the construction of the interactive sunburst. Since the genes are conventionally annotated with only the most specific GO term, we also created a set of functions to annotate them with all their parent terms in order to be able to query the relevant subset by using more general terms that encompass their functions. Both programs are available as supplementary files.

### Mabellini database

In addition to the modelled structures and functional annotation (e.g. GO terms), Mabellini data are mapped to both Uniprot and Pfam and also track the predicted protein properties (including membrane protein topology and disorder). Membrane protein topology has been predicted with TOPCONS (59) metapredictor, including the signal peptide predictor as implemented in SPOCTOPUS (60). Disordered regions in the evaluated proteins have been assessed with DISOPRED (61). Both approaches were chosen for their robustness and prevalence in the field, as well as capability for a relatively high throughput.

The database itself has been deployed on a PostgreSQL server and was implemented using SQLAlchemy Python module. The database schema (Supplementary Figure S4) follows Codd’s third normal form (3NF), thus making for a more robust and future-safe representation.

### Website development

The website back-end is managed using the SQLAlchemy Python module, which allows for the direct access and query of the underlying database populated by Vivace. In order to decrease query retrieval time, and thus allow more users to use Mabellini DB at a time, we parallelized all the tasks using the Multiprocessing Python module. Furthermore, using a combination of publicly available application programming interfaces (APIs) and scripts developed in-house, we provide users direct mappings to other well-established database resources such as PDB, Uniprot, Pfam, SCOP and CATH.

The website is extensively based on the Flask module, which is used to generate the website pages and provide connectivity with the SQL database. The website is written in HTML5 using CSS, Javascript and JQuery as well as the Bootstrap (version 4) framework. JINJA2 templating language for Python was used to dynamically generate HTML templates. The sunburst charts were created using the D3.js package, using the information generated by Hiera-Go and Prote-GO algorithms in JSON format as data source.

In order to facilitate programmatic usage of the Mabellini database, significant fraction of data stored there has been made available through a RESTful API. Data is returned in form of JSON objects, thus is easily parsable by downstream software and straightforwardly incorporated into existing solutions.

## Results

### Structural proteome characteristics

We have built a comprehensive set of *M. abscessus* protein structures, covering 3394 out of 4940 protein coding sequences annotated in the UniProt UP000007137 proteome. This comprises 13 879 models, of which 1855 are bound to a ligand (cofactors, substrates, small molecules, etc. that are retrieved from the structures used as templates). Despite the paucity of experimentally determined protein structures of *M. abscessus,* with only 53 entries in the PDB, Mabellini now addresses 68.7% of gene products.

The models included in the final database had a median length of 211 residues, with the longest (MAB_2301c: Rank 01, a putative mmpL transporter) being 946 residues long. The average gene coverage of our models is 75.4% while the median gene coverage is 92.1%, showing that we have mostly generated near-complete proteins. The average Molprobity score was of 3.21 with a standard deviation of 0.42, when considering all models conjointly, but it is lower (3.15) when considering only non-liganded models and higher (3.56) when considering only the liganded models, likely due to the algorithm employed for the incorporation of ligands, as described in the methods section. The Molprobity score is a log-weighted value that takes into consideration atomic clashes, main-chain and side-chain features, and the resulting number represents the crystallographic resolution at which those features would be expected (54).

**Figure 3:**
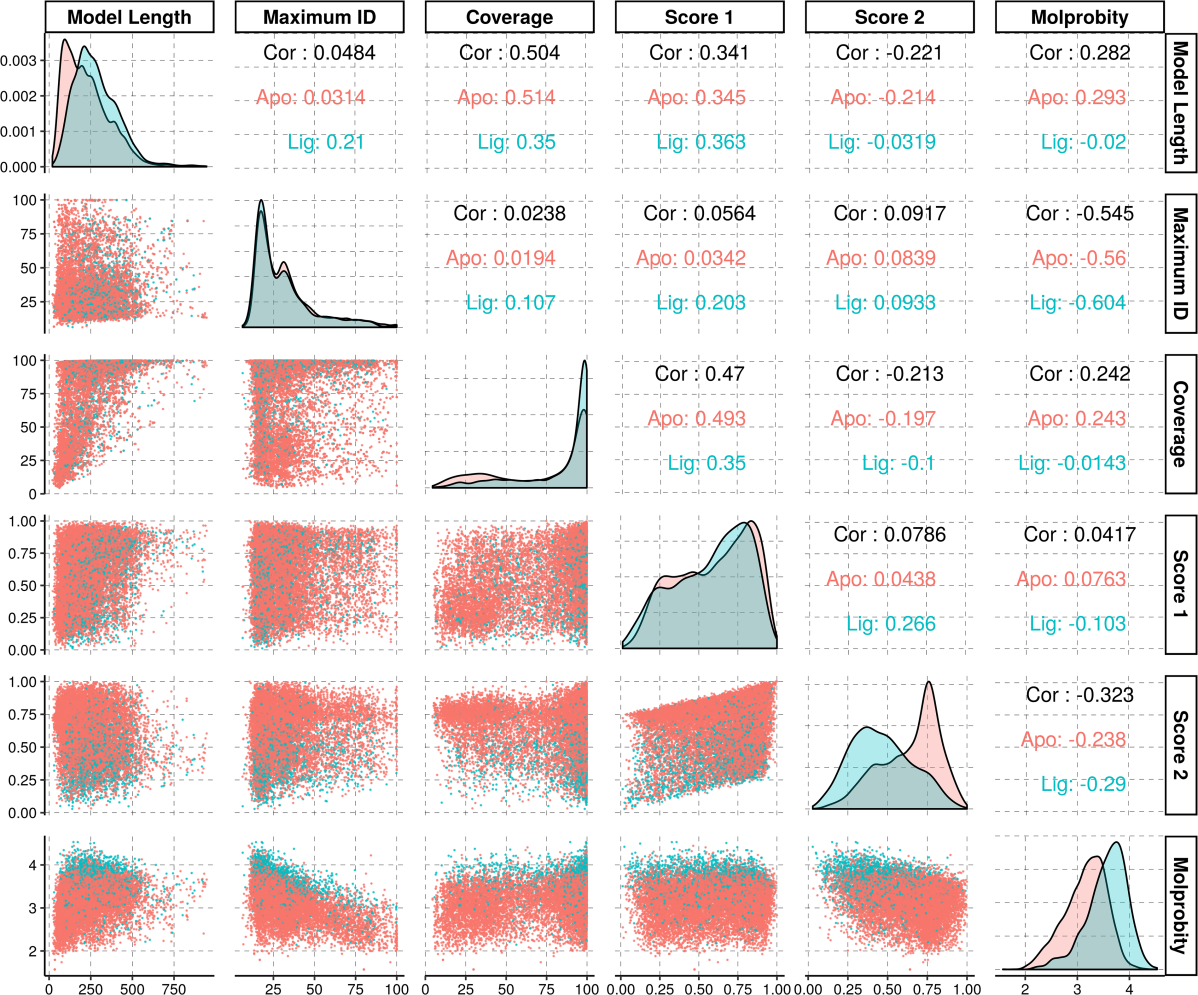
Density and scatter plots of the properties calculated for each of the models. The colours represent the presence (cyan) or absence (red) of a ligand. Density plots are shown in the diagonal representing the distribution of each individual property. The bottom half contains the scatter plots and the top half of the graph presents the correlation coefficients.

The average identity of the modelled protein sequences to the most similar template was of 31.7%, with a first quartile value of 18.2% and third quartile of 38%, yielding an interquartile range of 19.8%. As our approach is profile-based, some of the templates had identities significantly below the generally established limits for comparative modelling, and yet rendered models with structural features compatible with evolutionary domain annotations. Further statistical information regarding the models’ properties may be found in Figure 3.

Every produced model has been subjected to a multi-faceted quality assessment, using approaches described in the Methods section. Mabellini makes all of the produced models available for download, together with the quality assessment results; each of the models available in the website has the quality metrics embedded into the PDB file header, as well as a local quality estimate in the B-factor column.

### Modelled virulence factors

To illustrate some of the results generated by our pipeline, we selected a set of biologically relevant proteins involved either in important metabolic pathways or in antibiotic resistance. In order to be able to comment on the most likely functional state of a given target, we performed a simple PSI-Blast search to infer its oligomeric state and selected, for the following analysis, proteins that are likely functional in a monomeric state.

Table 1 summarizes the proteins presented in this section together with the source of evidence for their biological relevance and features of the best-ranking model generated for them.

**Table 1:** Features of the best models built for proteins selected from the literature

Name	Prot. len.	Model len.	Cov. (%)	Max. Id. (%)	Score1	Score2	Molprob.	Relevance	Reference
MAB_0591	141	137	97.2	64.5	0.722	0.616	2.861	Rifamycin Resistance	(22)
MAB_0935c	572	571	99.8	39.3	0.936	0.571	3.159	TPP Biosyntetic Pathway	(32)
MAB_0938c	486	432	88.9	15.3	0.836	0.811	3.347
MAB_1496c	475	474	99.8	32.4	0.853	0.442	3.817	Tetracycline Resistance	(22)
MAB_2297	173	172	99.4	23	0.845	0.48	3.861	Macrolide Resistance	(14), (22)
MAB_2385	255	254	99.6	32.2	0.963	0.837	2.87	Aminoglycoside Resistance	(22)
MAB_2875	289	245	84.8	99.6	0.504	0.71	2.258	β-Lactam Resistance	(22)
MAB_4100c	76	75	98.7	82.7	0.451	0.415	2.809	GPL Biosynthetic Pathway	(64)
MAB_4103c	267	225	84.3	20.2	0.6	0.381	4.024		
MAB_4105c	262	236	90.1	23	0.433	0.758	3.587		
MAB_4108c	268	262	97.8	47.2	0.683	0.312	3.041		
MAB_4109c	230	229	99.6	28.3	0.888	0.643	3.567		
MAB_4111c	353	348	98.6	31.2	0.926	0.923	3.345		
MAB_4112c	440	418	95	19.3	0.96	0.684	3.44		
MAB_4113	288	287	99.7	60.1	0.887	0.833	2.651		
MAB_4115c	987	331	33.5	16.3	0.82	0.405	3.421		
MAB_4116c	959	939	97.9	13.5	0.964	0.567	3.548		
MAB_4117c	138	89	64.5	50	0.778	0.865	2.954		

**Figure 4:**
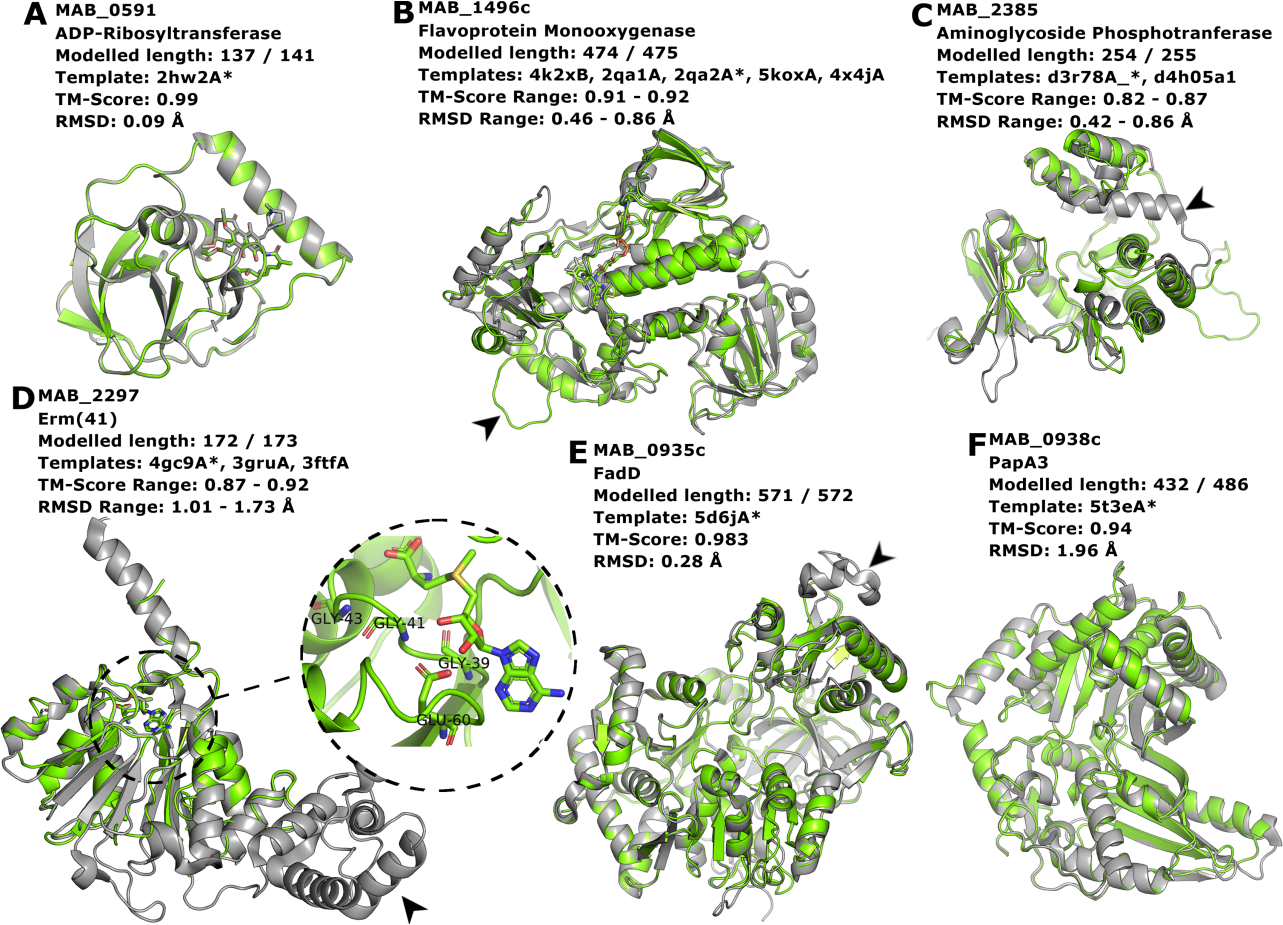
Interesting targets selected from the literature. (**A**–**C**) Antibiotic modifying proteins revised in (22). (**D**) Erythromycin ribosomal methylase (Erm (41)) with the SAM binding pocket and the GxGxG motif in detail. (**E**–**F**) FadD and PapA3, enzymes likely involved in the TPP-biosynthesis pathway as suggested in (32). In all cases, the models are represented in green and are superimposed to the most dissimilar template (i.e. the one presenting highest RMSD value), which is also indicated in each header with an asterisk. The black arrowheads point to different features described in the main text.

Proteins MAB_0591 (Figure 4A), MAB_1496 (Figure 4B) and MAB_2385 (Figure 4C) have been recently reviewed in (22) and are involved with resistance to the antibiotics rifamycin, tetracycline and aminoglycosides, respectively.

MAB_0591 (Arr_Mab) is an enzyme that catalyses the adenosine diphosphate (ADP)-ribosylation of rifamycin antibiotics, thus conferring resistance to this class of compounds. The 137 of the 141 residues in this enzyme were modelled, yielding a model with a Molprobity score of 2.861 and high structural similarity to the used template from *M. smegmatis* (CATH ID: 2hw2A00, TM-score: 0.99, RMSD: 0.09 Å). We were able to model the liganded state, in which it is complexed to rifampicin. As this model is built based on a very close homolog, it has a high expected verisimilitude, which means it could plausibly serve as a target in molecular docking (and re-docking) studies.

MAB_1496c encodes a flavoprotein monooxygenase implicated in resistance to tetracyclines. Interestingly, among the flavoprotein templates selected, a rifampicin monooxygenase from *Nocardia farcinica* (PDB ID: 5KOX) shares 31.9% identity with the *M. abscessus* sequence. The top-ranked model is bound to the FAD cofactor. Superimposition of all the templates onto the Rank 01 model yields a maximum RMSD of 0.86 Å, which indicates a largely structurally conserved protein, considering that the sequence identities range from 29.9% to 32.4%. When compared to all templates, MAB_1496 presents a 12-residue-long insertion at position 76, resulting in a longer loop formed between helices 3 and 4 (Figure 4B, black arrowhead), near the substrate-binding site, which could be related to interaction specificity.

MAB_2385 codes for an 3′′-O-phosphotranferase, which transfers a phosphate group from an adenosine triphosphate molecule to the N-methyl-L-glucosamine moiety of streptomycin and is implicated in resistance to that compound. All models generated for this protein have coverage of 99.6% and the best models’ RMSD values to the templates range from 0.42 to 0.86 Å. The most notable difference between the model and templates is the absence of a C-terminal α-helix in our model (Figure 4C, black arrowhead).

The protein Erm (41), encoded by the MAB_2297 gene, is one of the main effectors for clarithromycin resistance. Despite having low sequence Identity to the templates used in the modelling (17.3–23.3%), the generated models cover 93.6–99.6% of the protein length, and exhibit a correct topology, bearing a canonical Rossman fold domain. The Rank 01 model is bound to S-adenosylmethionine (SAM, AdoMet), which is a cofactor for class I methyltransferases. All models contain the characteristic nucleotide-binding GxGxG motif (62) at the end of the first β-strand and the acidic residue GLU60 at the end of the second β-strand, which interacts with the SAM molecule via the hydroxylic groups of the ribose moiety (Figure 4D, in detail). The templates employed in the construction of the Rank 01 model bear an extra helical C-terminal domain, which is likely related to substrate specificity, (63) and is lacking in MAB_2297 gene (Figure 4D, black arrowhead).

Besides antibiotic resistance, we have also chosen to use as examples proteins involved in biosynthetic pathways that determine some important phenotypic aspects, such as the production of GPL and TPP.

Recently, TPP was suggested as the likely candidate for the formation of cord-like structures in *M. abscessus* and a specific genomic locus, which comprises the genes MAB_0934, MAB_0935c, MAB_0936c, MAB_0937c, MAB_0938c and MAB_0939, was identified as being responsible for the TPP biosynthesis (32). MAB_0935c codes for the FadD protein, an acyl-CoA syntethase, which is thought to activate and transfer C16 saturated fatty acids to the polyketide synthase (Pks) enzyme MAB_0939. Our best model for the MAB_0935c protein used PDB 5D6J as template, which is the FadD32 from *M. smegmatis*. The most noticeable difference between the two proteins is a large insertion in FadD32 from *M. smegmatis* when compared to MAB_0935c, which occurs between THR491 and GLU492 of our model (Figure 4E, black arrowhead). Nevertheless, the RMSD between the two structures was of 0.28 Å.

Also involved in the production of TPP is the PapA3 protein (Figure 4F), encoded by MAB_0938c, whose function consists of transferring the fatty acyl group elongated by Pks onto trehalose. Despite very low sequence identity (15.3%), the TM-score between the best model and the 5T3E template was of 0.94, indicating an excellent superimposition of the two structures. Both MAB_0938 and the 5T3E PDB template have been annotated with the Pfam domain PF00668, thus increasing the confidence of the inferred fold topology.

Another important genomic feature described in (64) is the GPL locus, comprising genes that range from MAB_4097 to MAB_4117. We were able to generate models for 11 out of the 21 genes present in this locus (two of which are putative proteins): MbtH-like protein (MAB_4100c), Fmt (MAB_4103c), Rmt3 (MAB_4105c), Rmt4 (MAB_4108c), Rmt2 (MAB_4109c), RmlB (MAB_4111c), Gtf3 (MAB_4112c), RmlA (MAB_4113), MmpL4b (MAB_4115c), MmpL4a (MAB_4116c) and MMpS4 (MAB_4117c) with coverages for the best models in each case ranging from 33.5% to 99.7%.

### Web-page front end

**Figure 5:**
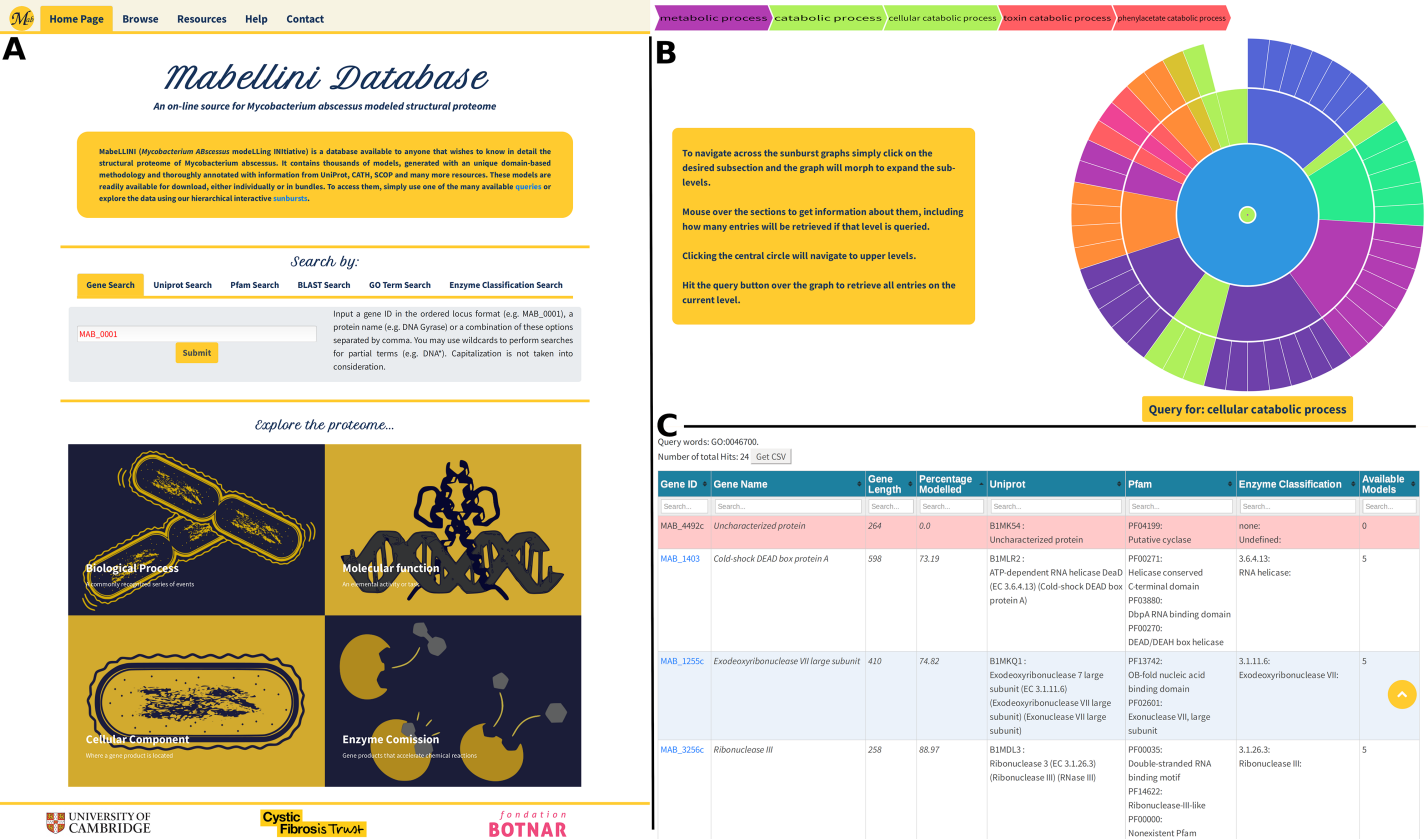
(**A**) Home Page, showing all query options and providing links to the sunburst navigation pages. (**B**) Sunburst representation of the Gene Ontology terms available for the *M. abscessus* proteome. The data were retrieved from UniProt and hierarchically classified using HieraGo. The graph is fully interactive and poses as an alternative querying system. Such classifications are available for the cellular component, molecular function and biological process ontology terms, as well as for Enzyme Commission numbers. (**C**) Searchable results table, showing information about the genes recovered by the initial query. Genes for which no models were produced are highlighted in red.

The website is accessible at the following URL: https://www.mabellinidb.science. For simplicity, the first page presents all types of queries available to the user (Figure 5A). By selecting the appropriate tag (Figure 5A top panel), the user has the option to search directly the *M. abscessus* proteome by: gene id, Uniprot id, Pfam id, sequence similarity, GO id and Enzyme Commission id (EC). However, the end user may not have prior knowledge of such terms, hence for each search field we implemented an additional text (keyword) search. For example, the RNA polymerase-binding protein (RbpA) has the Uniprot ID: ‘B1MAL8’; to retrieve all the genes with this Uniprot ID one can either directly input the exact id or use terms such as: ‘RNA polymerase’, ‘pol’ or ‘RNA’.

Furthermore, to make it even more straightforward for the end user to navigate the *M. abscessus* proteome, we created four sunburst queries (Figure 5A, bottom panel) based on GO terms and EC numbers. Figure 5B presents the sunburst for biological process. Here, users can click and hover over each segment of the sunburst. For example, if one is interested in genes involved in ‘alcohol metabolic processes’, he/she will first select ‘metabolic process’, then ‘small molecule metabolic process’ and finally ‘alcohol metabolic process’.

This allows the user to intuitively explore the hierarchical architecture we implemented. Moreover, at the top of the page, a breadcrumb trail (Figure 5B) will present each selection (click or hover) to inform the user about his/her position on the sunburst.

Once the desired level is achieved, the user can click the ‘Query: term’ button that will lead to the Multiple Results page (Figure 5C). In this page, the user is provided with a fully searchable summary table presenting all the hits as well as the query terms used, the total number of genes retrieved and a ‘Download’ button. In the summary table for each entry, we provide the gene’s id (ordered locus), name, length, the percentage modelled by our algorithm, its Uniprot and Pfam ids, the EC codes (when available) and the number of models generated. The genes for which no models were produced (due to the lack of suitable templates) are highlighted in red in the query results table. For all other entries, the user can access the models by selecting (clicking) the gene id of interest.

**Figure 6:**
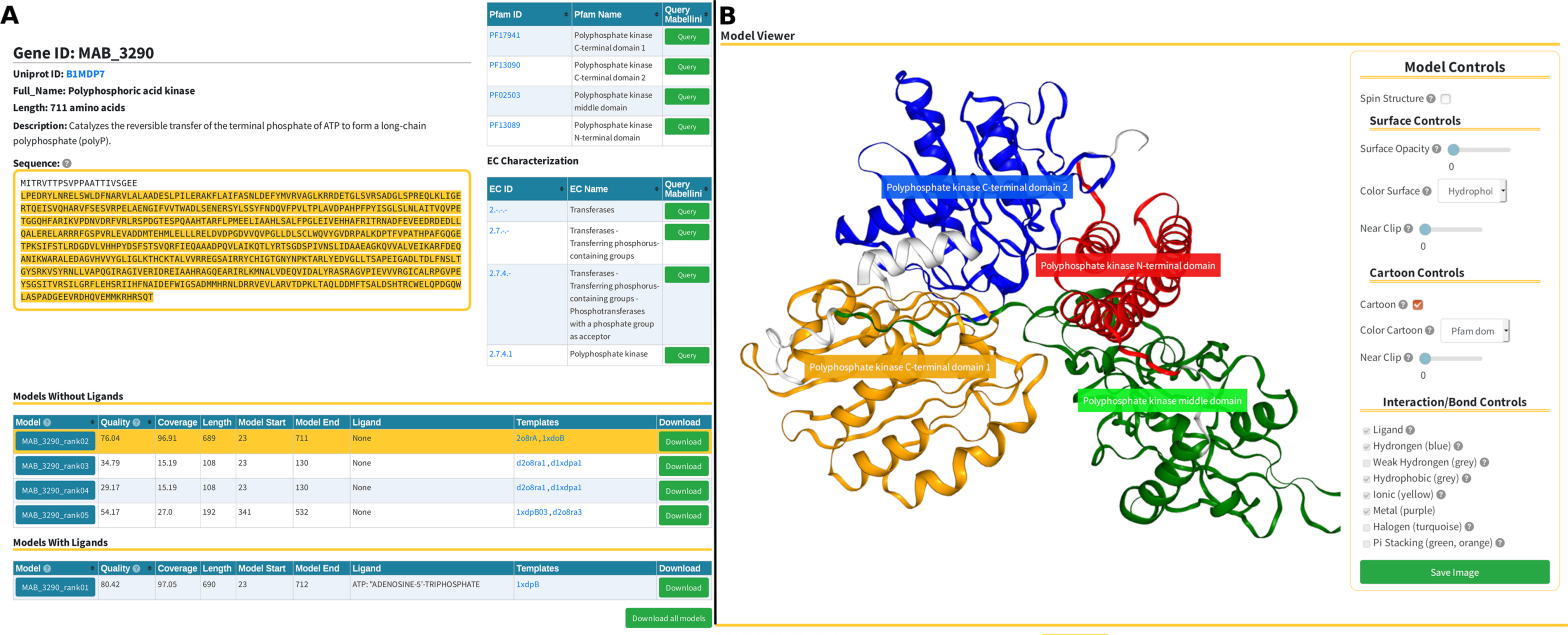
Models page. (**A**) Information about the selected protein in the top section (UniProt ID, enzyme commission number, Pfam domain, protein sequence and description), followed by tables with the models’ attributes for both liganded and non-liganded models. (**B**) In the bottom section, an NGL model viewer is displayed with a number of interactive options. The models are available for direct download, both individually or as a bundle.

The Models page (Figure 6) is divided into three sections. At the top, we present background information about the gene of interest, as well as mappings to Pfam domains and EC classification (Figure 6A, at the top). In the following section, we display a list of generated models split in two tables that reflect the model’s liganded state (Figure 6A, at the bottom). The tables contain the models’ qualities, coverage, length, residue interval and the templates used in the modelling process. If there are experimental structures available, they are also displayed as an additional table at the top of this section. The last section presents an interactive panel, built using NGL, where the user can inspect any model selected in the tables presented above (Figure 4B). For example, using the Model Controls the user can display the surface contour, colour by different properties or according to the Pfam domains, view the ligand interactions, save an image file, etc. Additionally, the user can download the structures by clicking the ‘Download’ (for individual entries) or ‘Download all models’ (for batch download) buttons.

The implementation of a RESTful JSON-based API in Mabellini increases its utility for the advanced user intending to incorporate the database in their software solution. Through a set of powerful, yet simple, query mechanisms (see Supplementary Material and description in the website), one may selectively query the database and retrieve the desired subset of data stored therein. Due to a low-level implementation, sharing the query engine with the website, this API is both fast and robust, permitting for a substantial traffic and self-limiting in cases of excessive usage. Even though website and API share the same engine, they are mutually independent, thus performance of one is not fully contingent on the other.

In summary, we have created an intuitive and functional website that should be of interest both to researchers interested in the biological aspects of *M. abscessus* and those generally interested in protein structure prediction and proteome modelling.

## Discussion

Genomic and proteomic databases are becoming increasingly widespread. Presently, there are multiple online services available, such as the GenBank (65), UniProt, KEGG (66) and Ensembl (67) databases, in which it is possible to find extensive information regarding the gene products. Bacteria-specific databases have also been developed in the past decades, as is the case of BacMap (68), a genome atlas where one can explore the complete genome of over 1790 bacterial species. These more specific databases usually have a more direct approach in the data presentation, normally best suited to the target group of users. The Mycobrowser service, for example, contains genomic information for eight different mycobacterial species, including *M. leprae*, *M. tuberculosis* and *M. abscessus*. It provides tools to explore the whole-genomes of these microorganisms, including summarized genome data and the sequences of individual genes. Nevertheless, these databases are usually dedicated to the curation and displaying of protein and gene sequences and few of them tackle the three-dimensional structure of proteins.

Some of the most recent resources, such as the Genome3D (69, 70), ModBase and even the COSMIC (71) database (the Catalogue of Somatic Mutations in Cancer), have invested in the three-dimensional representation of proteomic data. Genome3D is a recently established collaborative database that aims to coalesce structure and sequence-based annotations, as well as three-dimensional models, in a centralized hub, providing consensus annotations for a number of model organisms (currently 10 different organisms, including *Homo sapiens, Caenorhabditis elegans, Escherichia coli, Saccharomyces cerevisiae* and *Drosophila melanogaster*). Progress is underway to integrate the ‘Vivace’ pipeline as an additional modelling source.

Chopin, the *M. tuberculosis*-related resource based on Vivace pipeline, released by our group in 2015, has attracted interest. Since then, we have rectified its observed shortcomings in terms of template availability, template selection and model selection. We have updated our underlying database (TOCCATA) to reflect the current state of knowledge and instituted protocols for nearly fully automated updates in the future. In comparison to the TOCCATA version used to build the original CHOPIN, the new version contains nearly twice the amount of data, with 10 842 single-domain profiles and 4162 multi-domain ones, comprising over 604 000 structural templates (PDBs), while the older version contained 6590 and 2261 single- and multi-domain profiles and ~314 000 structures. Additionally, the new TOCCATA contains 12 103 ‘unclassified’ profiles, containing structures with no SCOP/CATH domain assignments, to be searched in case of lack of satisfactory hit among the single- and multi-domain profile sets. We have also optimized the parameters for the template selection, allowing for a higher expected quality of predictions. Finally, we have also conducted a sanity check and enforced modelling using the ‘unclassified’ profiles that contained structures identified by a BLAST search against PDB.

Since our approach is mainly based on comparative modelling, although we have been able to generate models for roughly 75% of the *M. abscessus* proteome, we are still restricted by structural data available in public repositories for close homologues. In our experience, structural information about complex multi-domain gene products is especially scarce, a tendency that is likely to change as a result of the recent advances in alternative methods for protein structure determination, such as cryo-EM. *Ab initio* methods for protein modelling, on the other hand, are still not reliable enough to be incorporated in databases like Mabellini, since the usefulness of the resulting models would be questionable. Therefore, endeavours such as the National Institutes of Health-funded Protein Structure Initiative and the several structural genomics projects (72) remain of paramount importance in enabling the generation of high-confidence comparative models.

Furthermore, Mabellini is based on TOCCATA, which is inherently oriented toward evolutionary domains. This yields models that often do not span the entire protein. While for many proteins we have produced well-substantiated models of multi-domain proteins based on multi-domain homologues, there is still room for improvement where there is no homologue. We intend to address this aspect in the upcoming updates to the methodology and to the resource itself.

The in-depth analysis of some biologically relevant targets showed that the models used to populate Mabellini exhibit a correct fold and satisfactory stereochemistry, thus being highly valuable. They may serve a number of distinct purposes that include, but are not restricted to general structure investigation, understanding function and selectivity, target fishing, molecular docking, virtual screening, molecular dynamics and the analysis of the impacts of mutations.

## Conclusion

Mabellini, the comprehensive resource for theoretical models of *M. abscessus* proteins is, to the best of our knowledge, the first resource of its kind for this particular pathogen. While species-specific databases for bacteria have been developed for decades, they predominantly focus on genomic characteristics, instead of structure and function.

The methodology used in the construction of Mabellini is a major advance over the one used for Chopin. While the underlying rationale remains largely the same, we have improved homology detection and introduced multi-domain and full-chain (‘unclassified’) profiles into database. The ‘fast track’ link between PDB and TOCCATA (through an initial BLAST search) ensures we do not miss any of the close-to-native structures.

We have built an entirely new, streamlined web-based user interface for Mabellini, which allows for an easy access to the data, with special focus on usability and ease of access to information.

Besides performing regular updates, our goal is to also aggregate to the database functional annotations and protein–protein interactions derived from publicly available sources, pathway analysis (using databases such as BioCyc (73)), assessment of effects of mutations through state-of-the-art methods developed by our group (SDM (74) and mCSM (75) family of methods), as well as genome-wide coupling analysis results (76). Efforts are underway towards the development of robust methods to model accurately the structure of multimeric proteins. We want to move beyond simple comparative modelling to allow for constructing and accurately selecting correct assembly modes.

## Supplementary Material

SupplementaryMaterial-Revised_baz113
