# Supplementary material for: Mabellini: a genome-wide database for understanding the structural proteome and evaluating prospective antimicrobial targets of the emerging pathogen Mycobacterium abscessus
Source: Database (Oxford). 2019 Nov 13;2019:baz113. doi: 10.1093/database/baz113 (PMC6853642; doi:10.1093/database/baz113)
Supplement: SupplementaryMaterial-Revised_baz113 [file supplementarymaterial-revised_baz113.pdf]

Mabellini: a genome-wide database for understanding the structural proteome and evaluating prospective antimicrobial targets of the emerging pathogen *Mycobacterium abscessus*

Supplementary Material

Marcin J. Skwark[1¶], Pedro H. M. Torres[1¶], Liviu Copoiu[1¶], Bridget Bannerman[1], R. Andres Floto[2,3] and Tom L. Blundell[1*]

[1]*Department of Biochemistry, University of Cambridge, Cambridge, CB2 1GA, UK*

[2]*Molecular Immunity Unit, Department of Medicine University of Cambridge, MRC-Laboratory of Molecular Biology, Cambridge, CB2 0QH, UK*

[3]*Cambridge Centre for Lung Infection, Royal Papworth Hospital, Cambridge, CB23 3RE, UK*

*Corresponding Author
E-mail: tlb20@cam.ac.uk (TLB)

[¶]These authors have contributed equally to this manuscript

AEROSPACI scores supplementation

Both VIVACE and TOCCATA are fundamentally based on a SCOP+CATH combination of domain annotations and metadata. One of these are AEROSPACI scores present in SCOP. Nevertheless, a significant fraction of structures present in PDB at the time of preparation of this work were devoid of AEROSPACI scores. To circumvent this, we have devised a simple, linear model:

$$A_s(\boldsymbol{x}) = \sum_{i=0}^{4} \beta_i x_i$$

where x designates observed values, β designates the fit parameters (see below) and indices correspond respectively to: resolution, reciprocal of resolution, *rvalue*, reciprocal of *rvalue* and ratio of resolution to *rvalue*. We have used the values present in the SCOP "aerospaci-all-data-2018-05-17.txt" file and applied a grid search algorithm to find the best set of hyperparameters for the Lasso algorithm using the scikit-learn python module. For hyperparameters of $\alpha = 1e\text{-}5$, $max\_iter = 5000$, we found that parameters $\beta = [-0.04354, 0.91651, -1.03199, 0.00031, -0.00048]$ yielded the best goodness of fit to observed values ($r^2 = 0.956$). We explored other regression methods (Ridge Regression and simple linear regression with stochastic gradient descent. The hyperparameter search was performed in a randomized, five-fold cross-validated regime.

The fit parameters have been applied to fill in the AEROSPACI values missing for certain PDBs.

API Usage

The currently implemented API includes queries by (i) Identifiers (Pfam, Ordered Locus, UniProt, Enzyme Comission, Gene Ontology), (ii) text, (iii) ligand ID, (iv) single model retrieval and (v) best models. The queries return JSON files that can be easily parsed programmatically containing information about the genes and models. The usage is described below.

Search by identifier:

Each of the identifiers is case insensitive. A query takes a single identifier and returns the information about gene (or genes) and links to the model JSON files.

**Usage**
http://mabellinidb.science/api/gene/[ID] - for ordered locus names
http://mabellinidb.science/api/oln/[ID] - alias for the former
http://mabellinidb.science/api/uniprot/[ID] - for UniProt IDs
http://mabellinidb.science/api/pfam/[ID] - for Pfam IDs
http://mabellinidb.science/api/EC/[ID] - for EC IDs
http://mabellinidb.science/api/GO/[ID] - for GO terms
**Examples:**
http://mabellinidb.science/api/gene/MAB_1234

Free text search:

Case sensitive search through the free-form fields of the database, that is names of genes and descriptions. The text is not tokenized, thus out-of-order search does not match.

**Usage**
http://mabellinidb.science/api/text/[text]
**Example**
http://mabellinidb.science/api/text/cell%20wall

Single model retrieval:

Given the internal model ID (returned by the search function above), return the URL of model's PDB and mmCIF files, as well as model descriptors in terms of quality, templates and ligand status.

**Usage**
http://mabellinidb.science/api/model/[ID]
**Example**
http://mabellinidb.science/api/model/MAB_1668__rank01__apo__1-341__Q_0.836_0.378.pdb

The function also recognizes queries in a form [ordered locus name]_[model rank]. The example below would retrieve the first ranked model for MAB_1234.

Example:
http://mabellinidb.science/api/model/MAB_1234__1

Retrieval of model IDs by ligand:

Given a three-letter ligand code (as per PDB small molecule dictionary), returns URLs for all the JSON records for models containing the ligand.

**Usage**
http://mabellinidb.science/api/ligand/[ID]
**Example**
http://mabellinidb.science/api/ligand/COA

Best models:

A convenience, parameter-free function, returning URLs of all the JSON records for the first ranked models.

**Usage**
http://mabellinidb.science/api/bestModels

Supplementary Figures

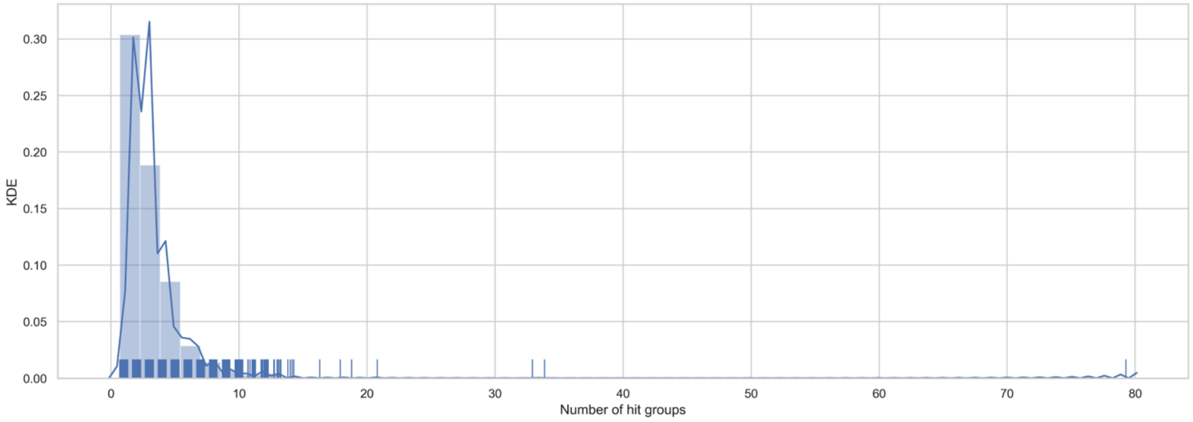

Figure S1 - The distribution of the number of TOCCATA hit groups among the Mabellini targets. For most of the targets, few (<5) hit groups have been identified, indicating a high confidence of the identified hits. Each of the ticks in the rug plot on the X-axis corresponds to the Mabellini target protein and the number of hit groups identified therein. Y-axis corresponds to the kernel density estimate. The evident outlier in the right-hand side of the plot is MAB_4691c (non-ribosomal peptide synthetase PstA) with 79 hit groups.

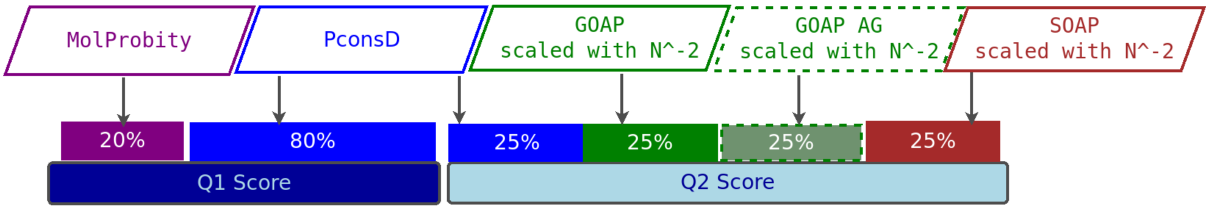

Figure S2 - Composition of Q1 and Q2 scores. Q1 is a linear combination of 80% structural consensus between models (computed by PconsD) and 20% stereochemical quality (percentage score). Q2 is a linear combination of GOAP-AG54, GOAP-score and SOAP55 potentials (scaled by the reciprocal of the square of number of residues in the model) and PconsD score. All four metrics composing the Q2 score have equal weights.

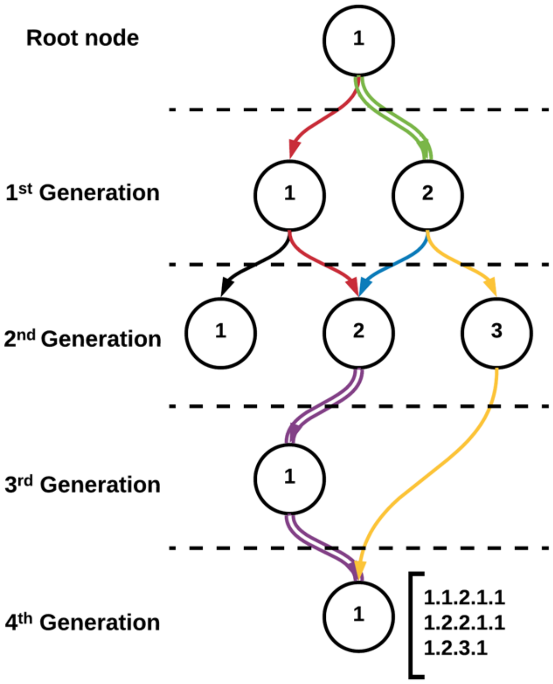

Figure S3 - Schematics of the hierarchical codes assigned to each GO term. Every node is assigned codes that represent every possible path from the root node. Also, the nodes in each layer (or generation) are numbered sequentially. These coded terms are then used to generate the hierarchical representation used in the interactive sunbursts (c.f. Figure 2).

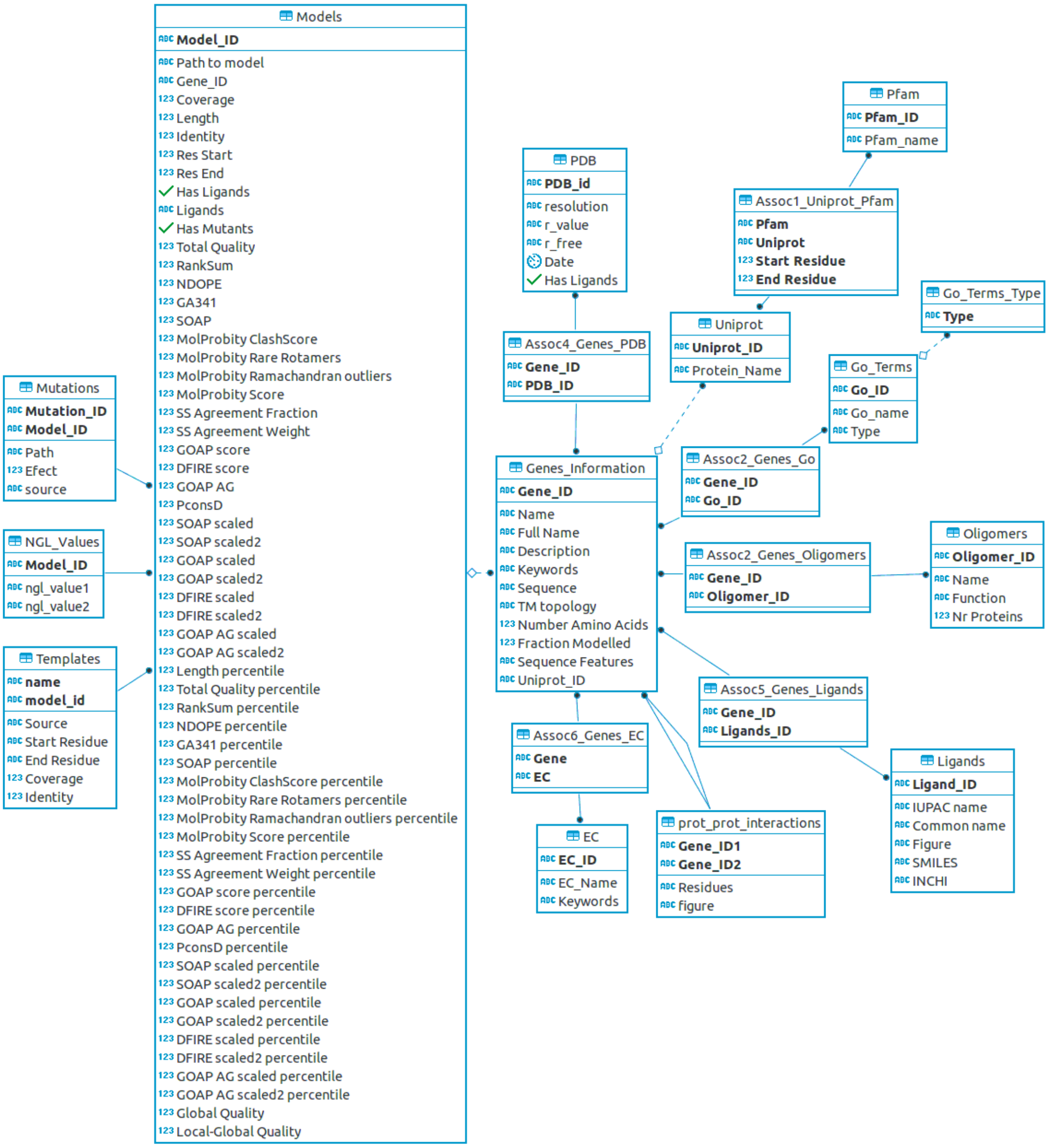

Figure S4 - Database Schema as implemented using SQLAlchemy. The *Genes Information* table consists the central hub connecting the bulk of the data. The models table comprises all the available information for the created models (quality, length, span, coverage, identity, etc.). A total of 5 association tables are in place to solve the many-to-many relationships (Uniprot-Pfam, Gene-GO Term, Gene-PDB, Gene-Ligands and Gene-EC). The NGL table contains information to be displayed in the NGL Viewer implemented on the website, such as the coordinates for the centres of mass of the Pfam domains. The database is already suited to receive the idealized updates, such as the effects of mutations and the oligomeric structures.
